# Oral Commensals in Healthy Individuals: A Clinicocytological Study

**DOI:** 10.7759/cureus.65317

**Published:** 2024-07-24

**Authors:** Nandhinipriya B, Gururaj Narayanarao, Sabarinath T.R, Rethika Singh B, Divyadharshini Chandrasekaran, Fadhila Rakeeba

**Affiliations:** 1 Oral and Maxillofacial Pathology, CSI College of Dental Science and Research, Madurai, IND; 2 Oral and Maxillofacial Pathology, CSI College of Dental Sciences and Research, Madurai, IND; 3 Department of Orthodontics, Sathyabama Dental College and Hospital, Chennai, IND

**Keywords:** personalized medicine, dysbiosis, symbiosis, commensals, microbiota

## Abstract

Background

Each human being has a specific group of microorganisms that are necessary for both sustaining health and causing illness. Normally, these microorganisms maintain bio-communalism, do not harm the host, and lead to a state known as symbiosis or eubiosis. The commensal nature of these bacteria is always maintained in symbiosis and attains pathogenic potential when there is an imbalance between host immunity and microorganisms. Our study focuses on the identification and differentiation of the various commensals present in the oral cavity of healthy individuals over a given period of time.

Aims and objectives

This study aims to: (i) identify various commensal bacterial species present in the oral cavity; (ii) differentiate each commensal bacterial species present in the oral cavity of healthy individuals using cytological and culturing methods; (iii) identify the presence of different types of commensal bacterial species in the same individuals with the specific time intervals; (iv) compare and correlate the presence or absence of bacterial species present as a commensal in both male and female; (v) identify and characterize the commensal bacterial species present in the oral cavity of healthy individuals; (vi) investigate the consistency of commensal bacterial species presence over time and between genders.

Methodology

We included sixty healthy individuals between the ages of 20 and 24 from both genders, took buccal smears once every two days for ten days, stained them with Gram stain, and grew them in blood agar and Mac Conkey agar.

Results

The most common commensals include Gram-positive cocci, and among them, Coagulase-negative staphylococcus species (85%) are predominant, followed by Staphylococcus aureus (13.33%), and Streptococcus species (1.67%). The presence of colonies remains the same in all three samples obtained from the same healthy individuals.

Conclusion

Loss of balance between commensals and pathogens can lead to dysbiosis, which results in disease.

## Introduction

Each human being has a specific group of microorganisms that are necessary for both sustaining health and causing illness. The organisms that have the beneficial effects of maintaining a symbiotic relationship within the body’s immune system are called commensals or symbionts [1]. In terms of cell count, a typical person harbors 90% bacteria as normal microflora, which inhabits the mucosal surfaces of the oral cavity. This ecosystem, with its unique atmospheric and nutritional demands, provides a symbiotic interaction with both the host and the ecosystem. Mucosal surfaces of the oral cavity, respiratory system, gastrointestinal tract, urogenital tract, and epithelial surfaces of the skin may contain these commensal bacteria [2]. The oral cavity harbors an average of 300 to 500 different bacterial species, including *Staphylococcus aureus*, *Haemophilus influenzae*, *Neisseria meningitides*, *Streptococcus pneumoniae*, Veillonella, *Actinomyces*, *Fusobacterium*, *Porphyromonas*, *Prevotella*, *Capnocytophaga*, *Lactobacterium*, and *Peptostreptococcus* [3]. The commensal nature of these bacteria is always maintained in a balanced state, and they attain pathogenic potential when there is an imbalance between host immunity and microorganisms. This condition is termed dysbiosis, which refers to the disease state, and the organisms causing this dysbiosis are called pathogens [4]. This alteration may affect the systemic condition and may produce illness. Long-term persistence of pathogenic microflora in the oral cavity may be a nidus for oral foci of infection, which in turn accounts for the systemic disease manifestations. Various general factors, such as diet, age, general health status, salivary PH, and salivary gland dysfunction, play a significant role in the transformation from symbiosis/eubiosis toward the state of dysbiosis [5]. A literature search reveals that disease acquisition requires certain conditions, such as host susceptibility, the local environment, an abundant number of pathogens, and their level of accumulation. Various authors in previous studies show that the most common commensal species include *Staphylococci*, *Lactobacilli*, and *Corynebacterium* species [6-11], and *Streptococcus* [10,12-18]. The objective of this research is to identify and distinguish among the diverse microbial species found in the oral cavity of healthy individuals at regular intervals for a period of ten days.

## Materials and methods

Sample collection

The approval for this study was obtained from the institutional ethical committee (ref: csicdsr/iec/0180/2021). A total of 60 individuals in the age range of 20 to 24 years were included in our study. A detailed case history and total oral health were recorded. The buccal mucosa of the subjects was scraped using a sterile cotton swab with mild pressure applied to it.

Sample categorization

The subjects were examined once every two days for a period of ten days. All the individuals had brushed twice daily in the morning and before sleeping at night. Scaling was done for all the individuals ten days before the commencement of the study. The smears were taken using a sterile cotton swab to identify and differentiate the oral commensals. The smear was prepared by spreading the cotton swab in a circular motion on a glass slide. It was then stained using the Gram staining procedure. The culture method was carried out using blood agar and Mac Conkey agar.

Inclusion criteria

Individuals who were apparently normal, had good oral hygiene, and were within the 20- to 30-year-old age groups were included in our study. All periodontal pockets should be less than 3 mm deep, and there should be no gingival irritation or redness. No dental caries or active white spot lesions should be present.

Exclusion criteria

Individuals with a known history of anemia or other nutritional disorders, diabetes, or a recent history of fever were excluded from our study. People who take antibiotics or use antiseptic mouthwashes regularly were also not included. Our study excluded individuals who regularly took inhaled steroids for asthma.

Stains and culture media used

For the cytological examination, the Gram stain was used to identify the presence of Gram-positive or Gram-negative bacterial species. The stained slides were analyzed for the identification of oral commensal bacteria under an oil immersion objective (×100) in a binocular compound microscope. For the purpose of culture, blood agar that is composed of sheep blood was used for the differentiation of the bacteria based on the type of hemolysis. Mac Conkey agar, composed of lactose monohydrate, sodium chloride, bile salts, neutral red, and crystal violet agar with peptone in semi-solid forms, was used to differentiate lactose-fermenting bacterial species from non-lactose fermenting bacterial species at 35-37 °C under aerobic conditions.

Statistical analysis

A descriptive type of statistical analysis was done using the statistical software Stata version 14 (StataCorp LLC, Texas, USA). Tables 1-2 provide demographic parameters, including age and gender, for the selected individuals. Fisher's exact test was used to find the association between the parameters, such as Gram stain and culture reports, taken at three different periods.

**Table 1:** Gender The data have been represented as N (%)

Gender	N (%)
Male	30 (50)
Female	30 (50)
Total	60 (100)

**Table 2:** Age categories The data have been represented as N (%)

Age categories	N (%)
20 years	21 (35%)
21 years	11 (18.34%)
22 years	13 (21.67%)
23 years	13 (21.67%)
24 years	2 (3.34%)
Total	60 (100%)

## Results

In our study, a comparison was made between the presence of commensals in healthy individuals by age, gender, and time period. A total of 60 individuals were included in our study. Both males and females were included, with an equal distribution of 30 cases in each group (Table 1).

Individuals were selected with an age range between 20 and 24 years. The proportion of individuals aged 20 years (35%) was higher than that of those aged 24 years (3.33%), as shown in Table 2.

The evaluation was done once every two days for a period of ten days, and five samples were taken in total. The cytological evaluation was done using the Gram staining method. The presence of Gram-positive cocci in clusters N (%) was shown as 45 (75%), 48 (80%), 51 (85%), 43 (71.67%), and 49 (81.67%), which were taken from days 1 (sample 1), 3 (sample 2), 5 (sample 3), 7 (sample 4), and 9 (sample 5), respectively. The presence of Gram-positive cocci in chains N (%) was shown as 15 (25%), 12 (20%), 9 (15%), 17 (28.34%), and 49 (81.67%) from day 1 (sample 1), day 3 (sample 2), day 5 (sample 3), day 7 (sample 4), and day 9 (sample 5), respectively. According to the Gram stain report, Gram-positive cocci represented in clusters were predominantly seen when compared with Gram-positive cocci in chains from all the samples (Table 3).

**Table 3:** Gram stain report The data have been represented as N (%)

Gram stain report	Sample 1 day 1 N (%)	Sample 2 day 3 N (%)	Sample 3 day 5 N (%)	Sample 4 day 7 N (%)	Sample 5 day 9 N (%)
Gram-positive cocci in chains	15 (25%)	12 (20%)	11 (15%)	12 (20%)	11 (15%)
Gram-positive cocci in clusters	45 (75%)	48 (80%)	49 (85%)	48 (80%)	49 (85%)
Total	60 (100%)	60 (100%)	60 (100%)	60 (100%)	60 (100%)

**Figure 1:**
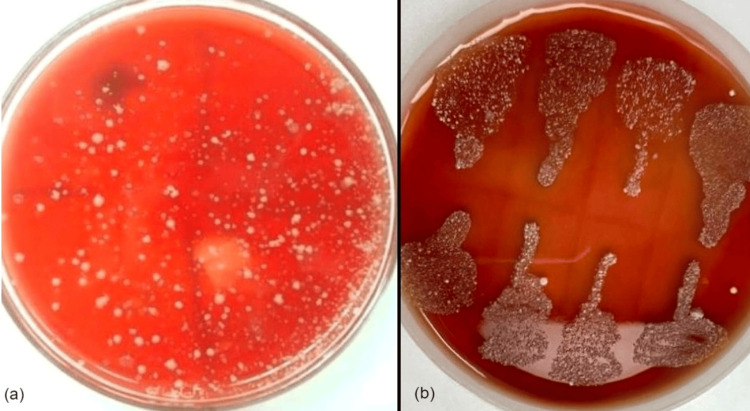
Culture methods (a) blood agar shows slightly raised small to medium sized hemolytic colonies with zones of alpha hemolysis, (b) Mac Conkey agar shows numerous, non lactose fermenting pale to white colonies

The culture was done using blood agar and Mac Conkey agar. Blood agar is used as a differentiating medium in the presence or absence of hemolysis. Mac Conkey agar is indicated only for the presence of Gram-negative species by differentiating between lactose-fermenting and non-lactose-fermenting colonies. After 18-24 hours of incubation done at 35-37 °C on blood agar, typical gray to white-colored, slightly raised, small to medium-sized hemolytic colonies appeared with zones of alpha hemolysis (Figure 1a). Numerous, slightly too well-raised, circular, varying size, non-lactose-fermenting pale to white colonies were seen in Mac Conkey agar, indicating the presence of coagulase-negative staphylococcus species (Figure 1b).

The presence of coagulase-negative *Staphylococcus* species N (%) was shown as 37 (61.67%), 42 (70%), 47 (78.34%), 39 (65%), and 42 (70%), respectively, in all five samples obtained. The Streptococcus species N (%) were seen as 15 (25%), 8 (13.33%), 4 (6.66%), 11 (18.34%), and 10 (11.66%), respectively, from all the samples obtained. The presence of *Staphylococcus aureus* was shown as 8 (13.33%), 10 (16.67%), 9 (15%), 10 (16.67%), and 8 (13.33%), respectively, in all five samples obtained. According to the culture report, the presence of coagulase-negative *Staphylococcus* species was seen predominantly in all five samples, followed by *Streptococcus* species and *Staphylococcus aureus* (Table 4).

**Table 4:** Culture report The data have been represented as N (%)

Culture report	Sample 1 (day 1) N (%)	Sample 2 (day 3) N (%)	Sample 3 (day 5) N (%)	Sample 4 (day 7) N (%)	Sample 5 (day 9) N (%)
Coagulase-negative Staphylococcus	37 (61.67%)	42 (70%)	47 (78.34%)	39(65%)	49 (81.67%)
Streptococcus species	15 (25%)	8 (13.33%)	4 (6.66%)	11(18.34%)	7(11.66%)
Staphylococcus aureus	8 (13.33%)	10 (16.67%)	9 (15%)	10 (16.67%)	4(6.66%)
Total	60 (100%)	60 (100%)	60 (100%)	60 (100%)	60 (100%)

The correlation was done between gender and each of the five samples' staining and culture reports.

Comparison of Gender and Gram Stain Report (Day 1, Sample 1)

The comparison was done between the Gram stain report of the first sample (day 1) and gender. It shows the presence of Gram-positive cocci present in clusters (78.34%) predominantly and shows a slightly higher quantity in females. Statistical analysis shows nonsignificant results of p-value 0.99 (Table 5).

**Table 5:** Cross tabulation for gender and Gram-stain report (day 1, sample 1) The data have been represented as N (%). p-value <0.05 is significant. F-Fisher's exact test. NS: not significant.

Gender	Gram stain report		Total N (%)	p- value^F^
	Gram positive cocci in chains N (%)	Gram positive cocci in clusters N (%)		
Male	5 (16.67%)	25 (83.34%)	30 (50%)	0.99 (NS)
Female	8 (26.67%)	22 (73.34%)	30 (50%)	
Total	13 (21.67%)	47 (78.34%)	60 (100%)	

Comparison of Gender and Gram Stain Report (Day 3, Sample 2)

The Gram stain report of the second sample (day 3) was compared to gender. It showed the presence of Gram-positive cocci present in clusters (93.34%), predominantly in males, and a slightly higher quantity in females. Statistical analysis showed a non-significant result (p-value = 0.86) (Table 6).

**Table 6:** Cross tabulation for gender and Gram stain report (day 3, sample 2) The data have been represented as N (%). p-value < 0.05 is significant. F-Fisher's exact test. NS: not significant.

Gender	Gram stain report		Total N (%)	p-value^F^
	Gram-positive cocci in chains N (%)	Gram-positive cocci in clusters N (%)		
Male	7 (23.34%)	23 (76.67%)	30 (50%)	0.86 (NS)
Female	2 (6.67%)	28 (93.34%)	30 (50%)	
Total	9 (30%)	51 (85%)	60 (100%)	

Comparison of Gender and Gram Stain Report (Day 5, Sample 3)

The comparison was done between the Gram stain report of the third sample (day 5) and gender. It showed the presence of Gram-positive cocci present in clusters (86.67%) predominantly and showed a slightly higher quantity in females. Statistical analysis shows non-significant results (p-value = 0.97) (Table 7).

**Table 7:** Cross tabulation for gender and Gram stain report (day 5, sample 3) The data have been represented as N (%). p-value < 0.05 is significant. F-Fisher's exact test. NS: not significant.

Gender	Gram stain report		Total N (%)	p-value^F^
	Gram-positive cocci in chains N (%)	Gram-positive cocci in clusters N (%)		
Male	11 (36.67%)	19 (63.34%)	30 (50%)	0.97 (NS)
Female	4 (13.34%)	26 (86.67%)	30 (50%)	
Total	15 (25%)	45 (75%)	60 (100%)	

Comparison of Gender and Gram Stain Report (Day 7, Sample 4)

The comparison was done between the Gram stain report of the fourth sample (day 7) and gender. It showed the presence of Gram-positive cocci present in clusters (76.67%) predominantly and showed a slightly higher quantity in males than females. Statistical analysis shows a non-significant result (p-value = 0.89) (Table 8).

**Table 8:** Cross tabulation for gender and gram stain report (day 7, sample 4) The data have been represented as N (%). p-value < 0.05 is significant. F-Fisher's exact test. NS: not significant.

Gender	Gram stain report		Total N (%)	p-value^F^
	Gram-positive cocci in chains N (%)	Gram-positive cocci in clusters N (%)		
Male	7(23.34%)	23(76.67%)	30 (50%)	0.89 (NS)
Female	10 (33.34%)	20 (66.67%)	30 (50%)	
Total	17 (28.34%)	43(71.67%)	60 (100%)	

Comparison of Gender and Gram Stain Reports (Day 9, Sample 5)

The comparison was done between the Gram stain report of the fifth sample (day 9) and gender. It showed the presence of Gram-positive cocci predominantly in clusters (93.34%) and a slightly higher quantity in females. Statistical analysis shows a non-significant result (p-value = 0.96) (Table 9).

**Table 9:** Cross tabulation for gender and Gram stain report (day 9, sample 5) The data have been represented as N (%). p-value < 0.05 is significant. F-Fisher's exact test. NS: not significant.

Gender	Gram stain report		Total N (%)	p-value^F^
	Gram-positive cocci in chains N (%)	Gram-positive cocci in clusters N (%)		
Male	9 (30%)	21 (70%)	30 (50%)	0.96 (NS)
Female	4 (13.34%)	28 (93.34%)	30 (50%)	
Total	13 (21.67%)	49 (81.67%)	60 (100%)	

Comparison of Gender and Culture Reports

The comparison was done between the culture report and gender. It shows the presence of coagulase-negative *Staphylococci* (86.67%) predominantly in males and a slightly higher quantity in females. Statistical analysis shows a non-significant result of p = 0.99 (Table 10).

**Table 10:** Cross tabulation for gender and culture report The data have been represented as N (%). p-value < 0.05 is significant. F-Fisher's exact test. NS: not significant.

Gender	Culture report			Total N (%)	p-value^F^
	Coagulase-negative Staphylococci N (%)	Streptococci species N (%)	Staphylococcus aureus N (%)		
Male	25 (83.34%)	1 (3.34%)	4 (13.34%)	30 (50%)	0.99 (NS)
Female	26 (86.67%)	0	4 (13.34%)	30 (50%)	
Total	51 (85%)	1 (1.67%)	8 (13.34%)	60(100 %)	

From our study result analysis, the presence of commensal bacteria in the samples taken at five different time periods shows no association with gender, Gram stain, or culture reports. As well, their association is not statistically significant with the P-value, which is >0.05.

## Discussion

Commensals refer to the minimal number of resident microbes, especially bacterial species. The most common commensals include *Lactobacillus*, *Bacteroides*, and *Staphylococcus*. They are involved in various functions such as digestion, maturation, and differentiation of the oral epithelium, regulation of the immune system by maintaining the balance between pro-inflammatory and anti-inflammatory mediators [18], detoxification of chemicals, and helping to maintain the functional barrier of the mucosal lining and skin [13]. According to Belkaid and Hand, Dekaboruah et al., and Khan et al., the most common commensal bacteria present include the *Staphylococcus* species, followed by *Streptococcus* and *Lactobacillus*. Our results were consistent with these studies [1,2,6]. These commensals prevent the entry of pathogenic bacterial organisms by blocking the mucosal surface of the body linings, secreting inhibitory substances such as antibacterial or antimicrobial agents, depleting nutrient substances, and down-regulating the virulence genes of pathogenic organisms [7]. According to Degruttola et al., Gao et al., and Deo and Deshmukh, the most common commensal bacteria was Lactobacilli [4,10,13]. Baty et al., Huo et al., and Irani show the presence of *Streptococci* [8,9,16] along with *lactobacilli* and S*taphylococci*, as the commonest commensal bacteria. It prevents the colonization of various pathogenic organisms, such as *Streptococcus pneumonia* and *Haemophilus influenza* [6,7,11]. In our study, samples taken on days 1, 3, 5, 7, and 9 show the presence of Gram-positive cocci, which is consistent with the above-mentioned studies. There is no difference in the presence of commensal bacterial species on those five days of examination. Our study shows the presence of Gram-positive Cocci in cluster form, predominantly according to the Gram stain results done with the samples. Further culture methods were employed using blood agar and Mac Conkey agar, and it was found that coagulase-negative staphylococci were predominant in all five samples obtained. These results were also correlated with a previous study done by Alghamdi in 2022 on the isolation and identification of oral bacteria and their characterization for bacteriocin production in the oral cavity. It maintains the state of symbiosis by producing bacteriocins that show anti-microbial properties that hinder the attachment and colonization of various microorganisms. If the balance between the commensal bacteria and pathogenic bacteria gets altered, it facilitates pathogenic growth and is referred to as dysbiosis [11]. Dysbiosis is a complex ecological shift in the oral microbiome involving alterations in microbial community composition, function, and host immune responses. It goes beyond a simple imbalance between commensals and pathogens [19]. Dysbiosis can also lead to dental caries, endodontic infection, periodontitis, alveolar bone loss, and other systemic diseases such as cardiovascular abnormalities, GIT, respiratory abnormalities, and diabetes [20]. Dietary supplementation with these commensal bacteria, such as *Staphylococcus*, *Bacteriodes*, and *Lactobacilli*, can be used for the prevention of dysbiosis [21]. This precision medicine can be used as a combined therapy, which paves the way for personalized, better treatment and a good prognosis for the individual patient. This study is the first of its kind to assess the presence of commensals in healthy individuals based on age group (20-24 years), specific period of time, and gender. Our study results show the presence of coagulase negative staphylococci remains unchanged when compared with these above-mentioned factors. Future studies on a large scale are required to confirm our study. Limitations of our study include a small range of age groups of individuals, such as 20 and 24 years, which restricts the generalizability of the findings. Dietary habits may differ from one individual to another, so standardization of dietary habits is required as it can alter the presence of microorganisms.

## Conclusions

This study provides preliminary evidence suggesting that coagulase-negative staphylococci may be a predominant commensal in the oral cavity of healthy individuals between 20 and 24 years. Bacteria are ubiquitous organisms and can be present in all individuals in certain amounts. Majorly, the loss of balance between commensals and pathogens can lead to dysbiosis, which occurs due to the increased amount of bacterial load in the oral cavity. Thus, the maintenance of eubiosis and the prevention of dysbiosis should be addressed to prevent disease manifestations. Dysbiosis and changes in the relationship with the hosts may determine the predispositions of systemic diseases. This non-invasive procedure of evaluation of oral commensals can be performed periodically to identify and eradicate the oral foci. However, due to the small age group, dietary habits, and lack of statistically significant associations, further research with larger cohorts is needed to confirm these findings and explore the potential implications for oral health. Maintaining eubiosis is essential for preventing dysbiosis and related oral diseases, and future studies should investigate the factors that influence the composition of the oral microbiome in healthy individuals.
